# Hypokalemic paralysis following intramuscular betamethasone injection: A case report and review of literature

**DOI:** 10.1002/ccr3.8923

**Published:** 2024-05-20

**Authors:** Pouya Ebrahimi, Homa Taheri, Seyed Ali Mousavinejad, Pedram Nazari

**Affiliations:** ^1^ Tehran Heart Center, Cardiovascular Disease Research Institute Tehran University of Medical Sciences Tehran Iran; ^2^ Cedars‐Sinai Cardiology Department Los Angeles California USA; ^3^ Lorestan University of Medical Sciences Khorram Abad Lorestan Iran; ^4^ Cancer Research Center Ahvaz Jundishapur University of Medical Sciences Ahvaz Iran

**Keywords:** acute neuromuscular paralysis, betamethasone, case report, hypokalemia, seasonal allergic rhinitis, severe adverse reaction

## Abstract

Acute neuromuscular paralysis is a relatively common condition in emergency rooms (ERs). They can be caused by several reasons, including adverse drug reactions. Betamethasone is a glucocorticoid commonly used for various conditions, such as allergic conditions. One of the rare but known side effects of glucocorticoids is hypokalemia. Rare cases of hypokalemia following high‐ and low‐dose glucocorticoid injections have been reported. This study presents the history of a young, healthy male without significant past medical history who presented with an inability to stand and walk due to four‐limb paralysis (more prominent in the lower limbs) following an intramuscular injection of a 4 mg betamethasone, which was prescribed for the treatment of allergic rhinitis. The patient was stabilized with an intravascular injection of potassium chloride diluted in 1000 mL of normal saline and monitored for 24 h, ruling out any other endocrine condition. Hypokalemia and its severe form are defined as the serum level of lower than 3.5 and 2.5 mEq/Lit, respectively. One of the etiologies of drug‐induced hypokalemic paralysis is systemic glucocorticoid administration. In severe cases, it can cause quadriplegia and other neuromuscular, respiratory, and cardiac complications. Therefore, it is an urgent condition that should be managed carefully. Pregnant women who are receiving these medications are a specific group at risk of hypokalemic paralysis. There are several safer treatments for seasonal allergic rhinitis compared to systemic glucocorticoids, which should be considered by physicians. Moreover, paralysis in patients receiving these medications should be approached attentively since it might be caused by hypokalemia, which can be life threatening if not treated. It is advisable that the blood level of electrolytes, especially potassium, be checked for patients who present with paralysis or weakness after glucocorticoid injections.

## 1 INTRODUCTION

One of the relatively prevalent neuromuscular complaints among patients presenting to the emergency rooms are rapidly progressive neuromuscular paralysis, which can reach its maximum level in less than days or weeks.^1^ A wide range of etiologies can cause this condition, and differentiating them is not always straightforward, but differential diagnoses can be limited by considering their specific features.^2^ These etiologies are generally categorized into four main groups, including: (1) anterior horn cell pathologies; (2) abnormal neuromuscular junction function; (3) peripheral nerve or nerve root dysfunction; and (4) muscular disease—periodic paralysis.^2^, ^3^, ^4^ Infectious, autoimmune, and toxic causes are the most prevalently seen causes of this condition, which can be fatal in some cases. Therefore, the reason should be identified, and an appropriate diagnosis should be initiated as soon as possible.^5^ One of the uncommon causes of neuromuscular paralysis is an abnormal serum potassium level.^6^

Potassium is an essential cation in the human body that regulates metabolic cell functions and muscle contractures.^7^ The abnormally low level of this ion (hypokalemia) is one of the rare but fatal causes of acute neuromuscular paralysis.^8^ This reversible type of paralysis can be induced by a few identified secondary causes, including drug‐induced hypokalemia.^9^ One of the medications that can cause this condition is glucocorticoids.^9^ Glucocorticoids are widely used for the treatment of various health conditions, such as allergic rhinitis. These medications can cause excessive renal loss of potassium and an increase in NA/K ATPase pumps, which results in the extracellular shift of potassium from skeletal muscles and consequently paralytic symptoms and hyperglycemia‐induced insulin secretion.^6^

This study presents a case of neuromuscular paralysis due to an abnormality in potassium level that manifested with progressive upper and lower limb paralysis, which was more prominent in the lower limbs. To the knowledge of the authors, this presentation of severe hypokalemia in a healthy young man following a low dose of glucocorticoid has rarely been reported in previous literature.

## 2 CASE PRESENTATION

### 2.1 Case history and examination

The patient was a 34‐year‐old male who had been brought to the emergency room (ER) with weakness and paralysis of the four limbs, which were more significantly observed in his lower limbs. His symptoms had started 8 h before his presentation and following an intramuscular injection of a 4 mg betamethasone, which had been prescribed due to allergic symptoms. The patient had rhinorrhea, itchy eyes, and pruritis in his face, which were not responsive to nasal corticosteroids. Therefore, an intramuscular injection of betamethasone was prescribed by his general practitioner the day before. The patient had no previous medical or drug history except nasal corticosteroid use due to his seasonal allergic rhinitis. There was no positive history of drug or alcohol abuse.

Based on the patient's ER form, in his physical exam, the patient was conscious and responsive, but he was unable to walk or stand. The vital signs were stable. The cardiovascular examination revealed no abnormalities, and in the neurology examination, except for lower limb paralysis, no significant abnormality was evident. Based on the Medical Research Council (MRC) scoring scale,^10^ his lower limbs strength was 1/5 (flicker or trace contraction) bilaterally and in the proximal and distal parts, while his upper limbs strength was 3/5 (active movement against gravity but not resistance) in both proximal and distal parts. He was not able to move his lower limbs actively and was unable to move his hands against resistance. Hypotonia, decreased deep tendon reflexes (DTRs), and flexor bilateral plantar response was also obvious in both lower limbs. Other neurologic examinations, including sensory nerves and sphincter function, higher mental functions, and cranial nerves, were intact.

### 2.2 Methods

His laboratory data, including complete blood count, comprehensive metabolic panel, blood electrolyte level, arterial blood gas, urine, and blood toxicology tests, including blood and urine alcohol levels, were unremarkable except for his serum potassium level, which was 2.5 mmol/L. Since the patient was a young man and had no renal or gastrointestinal risk factor for hypokalemia, endocrine causes of hypokalemia, such as hyperaldosteronism, 11‐beta hydroxylase, and 17‐alpha hydroxylase deficiency, and less common genetic disorders, namely apparent mineralocorticoid excess (AME) and glucocorticoid resistance syndrome, were considered in the history taking, physical exam, and laboratory evaluations. Plasma aldosterone to plasma renin activity ratio (ARR) for the exclusion of hyperaldosteronism, and 24‐h urinary cortisol accompanied by late‐night salivary cortisol for ruling out hypercortisolism were obtained. Even though the patient had received a glucocorticoid injection, all mentioned tests were unremarkable.

**FIGURE 1:**
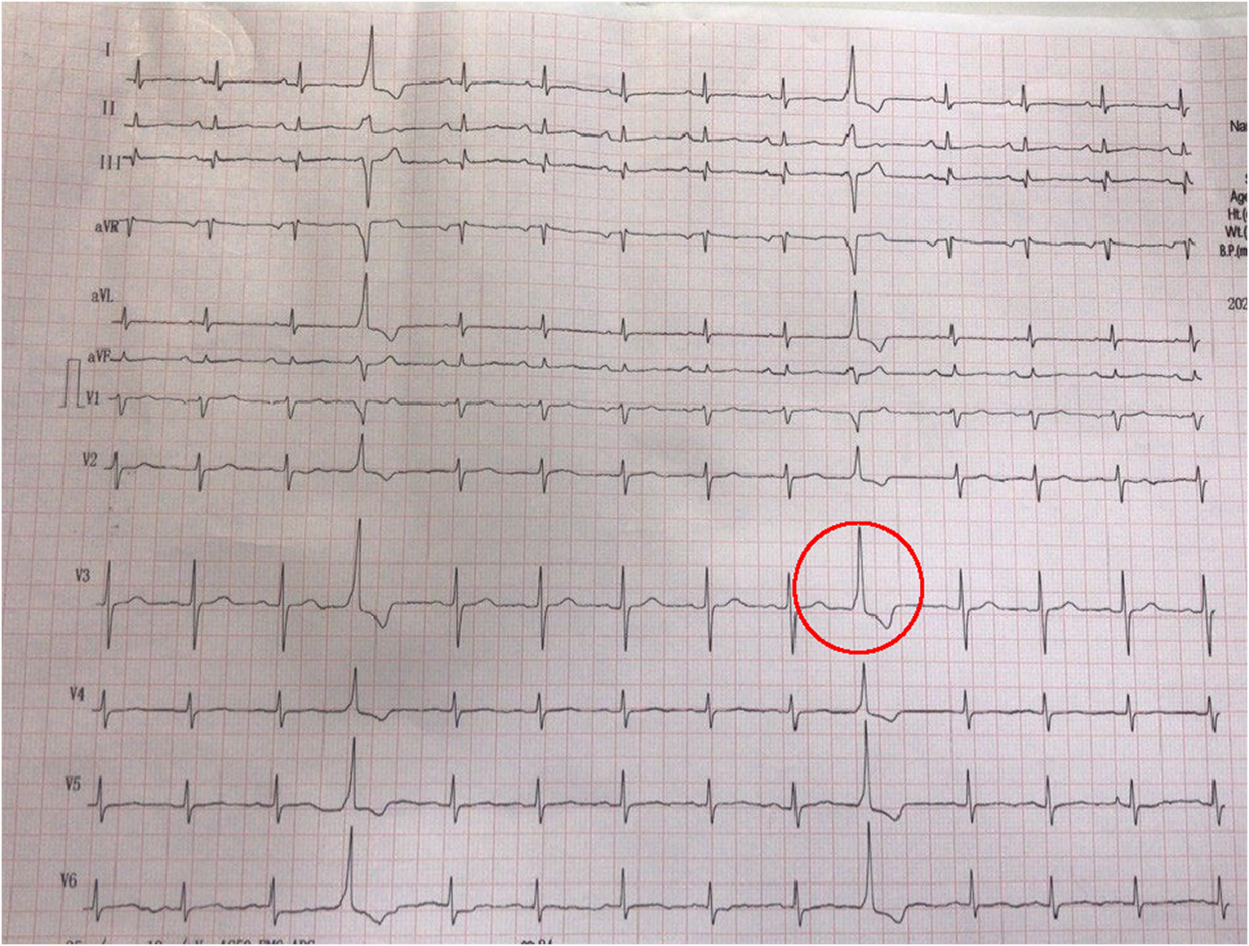
Electrocardiogram of the patient, when presented to the emergency room, was normal except for occasional premature ventricular contractions (red circle).

The patient's cardiac and respiratory functions were immediately monitored in the emergency room. The electrocardiogram showed no abnormality, except premature ventricular contractions (Figure 1). Based on neurological symptoms and hypokalemia (*K* = 2.5 mmol/L) in the results of blood samples, the intravenous treatment with potassium chloride (40 mmol KCl in 1 L 0.9% NaCl TDS) started immediately. After administration of potassium chloride diluted in 1000 milliliters of normal saline for 6 h, the patient's condition improved gradually, and the muscle weakness improved partially after 6 h. After being observed for 6 h in the ER, the patient was admitted for more observation during the next 24 h in the internal ward. All probable endocrine abnormalities that can cause hypokalemia were ruled out. The symptoms were completely resolved after 24 h of treatment.

### 2.3 Conclusion

Compatible with the medical team's expectation, the patient's condition remained stable without any further intervention or treatment. The patient was discharged the day after admission after making sure his serum potassium level is within normal range. Outpatient follow‐up 3 days, 1 week, and 1 month later showed no abnormality, and all laboratory and imaging studies were normal. The warning signs and probability of recurrence of symptoms and appropriate actions were mentioned to the patient. We advised him to note the event to his family physician in his medical appointments to prevent similar adverse effects occurrences, and this incident was recorded in his electronic file. In the Severe Adverse Reaction form, the name of the patient, his history and physical exam, the medication and the dosage, and the diagnostic and therapeutic interventions and their results were recorded.

## 3 DISCUSSION

Hypokalemia, defined as potassium levels lower than normal range (3.5–5 mEq per L),^11^ is mainly caused by decreased potassium intake, increased renal or gastrointestinal losses, or transcellular shifts into the cells.^12^ Severe hypokalemia is serum potassium level lower than ≤2.5 mEq/L (Table 1), which can present with different symptoms, including neuromuscular abnormalities (including muscle weakness and cramps), and respiratory or cardiac dysfunction, which might be detected by abnormal electrocardiography.^11^, ^12^ The emergence of any of the mentioned conditions necessitates urgent therapeutic intervention.^11^ Severe neuromuscular paralysis can occur due to hypokalemia caused by various reasons, including transcellular shift, gastrointestinal loss (diarrhea), abnormally high level of serum insulin and catecholamine, renal tubular acidosis (RTA), diabetic ketoacidosis (DKA), and hyperthyroidism. Dengue is another rare but reported cause of neuromuscular paralysis due to hypokalemia.^13^

**TABLE 1:** Severity, levels, and symptoms of hypokalemia.^21^, ^22^, ^23^

Severity	Level	Symptoms
Mild	>3.0–3.5 mEq/L	Mostly asymptomatic
Moderate	2.5–3.0 mEq/L	Abdominal cramping, malaise, myalgia, and weakness
Severe	<2.5 mEq/L	Changes in electrocardiogram such as ST‐segment depression, U‐wave elevation, and T‐wave inversion, arrhythmias, and paralysis

One of the relatively common causes of hypokalemia in young patients are endocrinopathies such as Cushing's syndrome, primary hyperaldosteronism, apparent mineralocorticoid excess (AME), 11‐beta hydroxylase and 17‐alpha hydroxylase deficiency, and glucocorticoids resistance syndrome.^11^ Patients with any sign or symptom suspicious for these diagnoses should be evaluated by plasma aldosterone to plasma renin activity ratio (ARR) for the exclusion of excessive aldosterone production. Furthermore, excessive glucocorticoid secretion and resistance of receptors to this hormone should also be ruled out by a 24‐h urinary cortisol, late‐night salivary cortisol, 1 mg overnight, or 2 mg 48‐h dexamethasone suppression test for suspicious cases.^14^, ^15^, ^16^, ^17^ Chronic alcohol use can also be a reason for hypokalemia and should be ruled out. This association is not well understood, but it has been attributed to more than normal gastrointestinal loss due to diarrhea and vomiting, excessive renal loss caused by renal tubular acidosis, and concomitant hypomagnesemia.^18^ The mechanism of hypomagnesemia‐induced hypokalemia is attributed to the increased movement of potassium to the extracellular space and distal tubule potassium excretion.^19^

Hypokalemic paralysis can also be caused by various medications, including antimicrobials (such as penicillin aminoglycosides, nafcillin, foscarnet, and ampicillin amphotericin B), beta2‐receptor agonists, diuretics, insulin, mineralocorticoids and glucocorticoids, laxatives, and xanthine.^7^, ^20^, ^21^, ^22^, ^23^, ^24^ There are various mechanisms for medications to cause hypokalemia, including increasing renal loss, which is the main mechanism seen in glucocorticoids, and antibiotic‐induced hypokalemia.^7^ Glucocorticoid agents are the known but rarely reported cause of hypokalemia via the extracellular potassium shift pathway. Increased Na+/K + ‐ATPase levels in skeletal muscle cells caused directly by glucocorticoids, along with mediating hyperglycemia with consequent insulin secretion^25^ and hypokalemia due to renal losses^26^ are the main mechanisms of glucocorticoids‐induced hypokalemia. Betamethasone‐induced hypokalemia was reported in 2011 in a pregnant woman by Corey M. Teagarden.^9^ In this case, a 23‐year‐old woman, gravida 3 para 2, presented at 32 weeks of gestation with leaking fluid and the diagnosis of preterm premature rupture of membranes (PPROM). She received two doses of 12 mg IM betamethasone 24 h apart, experiencing proximal limb muscle weakness after the first dose and exacerbation of symptoms and extension of that to neck and hip muscles after the second dose. The potassium was measured as 1.6 mEq/L. This condition is managed with intravenous potassium chloride.^9^ Severe hypokalemia is mainly treated by IV potassium chloride (10–20 mEq/h) diluted in 1000 mL of normal saline. The level of potassium, electrocardiogram changes, the way of infusion, which should be central for >10 mEq/h, and parallel oral potassium treatment should be considered during treatment.^22^ This study presents other case report literatures about hypokalemia in more detail (Table 2).

**TABLE 2:** Review of literatures for drug‐induced hypokalemia.

	Symptoms and signs	Laboratory data	Treatment and progression
Teagarden C, et al.^9^	A healthy 23‐year‐old gravid 3 woman at 32 weeks of gestation with preterm premature rupture of membranes received two doses of 12‐mg intramuscular betamethasone 24 h apart to accelerate fetal lung maturation. She developed significant proximal muscle weakness within 16 h after the initial dose	K: 1.6 mEq/L	Oral and parenteral potassium replacement restored her neuromuscular function over several days
Kulkarni M. et al.^32^	A 26‐year‐old multigravida at 36 weeks of gestation with gestational hypertension on treatment came with an acute onset of pain, numbness, and weakness of both legs, which worsened following betamethasone injection	K: 2.1 mEq/L	The patient's medical conditions were managed successfully with oral potassium supplements, antihypertensives, and regular monitoring of serum potassium levels until 37 weeks
Ryu S. et al.^25^	A 42‐year‐old man was admitted to the emergency department with upper and lower extremity paralysis. received 4 mg of betamethasone intramuscularly for urticaria at a local dermatology clinic	Na: 151 mEq/L, K: 2.4 mEq/L, ALD: 3.17 ng/dL Renin: 8.87 ng/mL/h	Potassium increased to 4.3 mEq/L after 24‐h replacement, and motor power improved progressively while maintaining normal potassium levels. On Day 6, muscle power recovered completely, and he was discharged
Ryu S. et al.^25^	A 34‐year‐old healthy man presented to the emergency department with acute‐onset muscle weakness. He had itching and erythema, for which he received 5 mg of dexamethasone intravenously, with symptomatic relief	Na: 147 mEq/L, K: 2.4 mEq/L, ALD: 7.12 ng/dL, PRA:0.49 ng/mL/h	Potassium levels normalized with motor improvement following continuous replacement. On Day 5, muscle weakness had disappeared completely, with no recurrent paralytic episodes to date
Rabi'at Muhammad A. et al.^33^	A 32‐year‐old grand multipara presented at 31 weeks gestation with numbness in all limbs for 9 days and a 1‐day history of weakness in all limbs. She had normal muscle bulk, with a power of 4/5 in both upper limbs and 3/5 in both lower limbs	K: 1.8 mmol/L, Na: 135	She had parenteral correction of potassium with complete resolution of weakness, and she was maintained on oral potassium supplements. She had an uneventful delivery at 37 weeks of gestation
Tahmasbi Sohi M, et al.^34^	A 30‐year‐old man with a medical history was significant only for chronic radicular low back pain. sudden‐onset quadriplegia 4 h after a transforaminal epidural steroid injection (TFESI)	K: 1.4 mmol/L, BS: 178 mg/dL. Serum bicarbonate: 16 mEq/L, Serum phosphorous level of 0.8 mg/dL	Muscle strength gradually improved with potassium supplementation
Tai H. et al.^6^	A 26‐year‐old with bilateral lower limb weakness received 5 mg of dexamethasone through intramuscular injection	K: 1.7 mEq/L, and CPK: 178 U/L Urinalysis was normal. However, the electrocardiogram showed a fattened T wave and exhibited U waves in the precordial leads V1–V3	Oral and IV potassium supplements with a total dose of 120 mEq were administered to the patient since the emergency department; hypokalemia resolved (serum potassium 4.0 mEq/L); and the patient could walk steadily

Abbreviations: AlD, aldosterone; BS, blood sugar (blood glucose); CPK, creatinine phosphokinase; K, potassium; Na, sodium; PRA, plasma renin activity.

Seasonal allergic rhinitis (SAR) is an IgE‐mediated, inflammatory process that is mainly triggered by environmental allergens. This condition involves the nasal mucosa, and the typical hint of its diagnosis is the presence of inflammatory cells within the mucosa and submucosa.^27^ The manifestation of this condition is mainly seen as nasal discharge, nasal itching, sneezing, and nasal obstruction.^28^, ^29^ The treatment of this condition is based on four main pillars: education of patients and their caregivers, avoidance of allergens and irritants, medications, and allergen immunotherapy in cases that are not responsive to previous steps.^27^ A remarkable point that should be considered by physicians is the fact that although glucocorticoid agents are one of the most effective agents in SAR treatment, there are several safer and more sufficient therapeutic options for the treatment of SAR, which include nasal corticosteroids, oral and nasal antihistamines, and topical ophthalmic products.^30^, ^31^

### 3.1 Clinical key point

Considering safety concerns related to the side effects of IM and IV injections of corticosteroids is an essential part of their prescription for any medical condition, and a history of any previous reaction should be obtained from the patient. There are several nonmedical and medical options for the treatment of SAR, and systemic glucocorticoids should not be considered the first line and routine treatment. Patients presenting with symptoms suspicious of hypokalemia, such as paralysis, weakness, and abnormal heart rhythm and ECG, should be evaluated for electrolyte levels of blood, especially potassium, as soon as possible. These patients' medical and drug histories should be obtained cautiously due to the necessity of ruling out endocrine disease or side effects of medications. Moreover, treatment of severe hypokalemia needs immediate action. However, the level of potassium should not rise excessively and without considering the method of IV administration, changes in potassium level, and cardiac function.

## AUTHOR CONTRIBUTIONS

**Pouya Ebrahimi:** Methodology; project administration; supervision; validation; visualization; writing – review and editing. **Homa Taheri:** Formal analysis; investigation; methodology; software. **Seyed Ali Mousavinejad:** Conceptualization; data curation; writing – review and editing. **Pedram Nazari:** Data curation; writing – review and editing.

## FUNDING INFORMATION

No funds were received for this study.

## CONFLICT OF INTEREST STATEMENT

The authors declare no conflicts of interest.

## CONSENT

Written informed consent was obtained from the patient to publish this report in accordance with the journal's patient consent policy.

## Data Availability

Further information regarding this study will be provided by the corresponding author on reasonable request.
